# Investigating modifications to participant information materials to improve recruitment into a large randomized trial

**DOI:** 10.1186/s13063-019-3779-4

**Published:** 2019-12-05

**Authors:** The HPS2-THRIVE Collaborative Group, Richard Haynes, Fang Chen, Elizabeth Wincott, Rejive Dayanandan, Michael J. Lay, Sarah Parish, Louise Bowman, Martin J. Landray, Jane Armitage, R. Collins, C. Baigent, Z. Chen, Y. Chen, L. Jiang, T. Pedersen, K. Rahimi, J. Tobert, P. Sleight, D. Simpson, A. Baxter, C. Bray, G. van Leijenhorst, Y. Mitchel, O. Kuznetsova, Richard Haynes, Fang Chen, Elizabeth Wincott, Rejive Dayanandan, Michael J. Lay, Sarah Parish, Louise Bowman, Martin J. Landray, Jane Armitage

**Affiliations:** 1 MRC Population Health Research Unit, Nuffield Department of Population Health, University of Oxford, Oxford, UK; 2 Clinical Trial Service Unit & Epidemiological Studies Unit, Nuffield Department of Population Health, University of Oxford, Oxford, UK

## Abstract

**Background:**

Large randomized trials are the best method to test the efficacy and safety of treatments expected to have moderate effects. We observed a significant decline in potential participants’ response to mailed invitations to participate in such trials over a 10-year period and investigated possible reasons behind this and potential modifications to the invitation process to mitigate it.

**Methods:**

Participants who declined to participate in the HPS2-THRIVE trial were asked to give a reason. Formal focus groups were conducted to explore the reasons that potential participants might have for not participating. In addition, two embedded randomized comparisons around the timing of provision of the full participant information leaflet (PIL) and its style were conducted during recruitment into this large randomized trial. HPS2-THRIVE is registered at ClinicalTrials.gov (NCT00461630).

**Results:**

The commonest reason given for declining invitations related to mobility and transportation (despite the offer of travel expenses). Both the focus groups and potential participants who declined their invitation indicated concern about side-effects of the treatment (as presented in the PIL) as a reason for declining the invitation. Neither delaying provision of the full PIL until the potential participant attended the trial clinic, nor modifying the style of the PIL improved the proportion of potential participants entering the trial: odds ratio (OR) 1.05 (95% confidence interval (CI) 0.94–1.17) and 1.10 (95% CI 0.94–1.28), respectively. However, modifying the style of the PIL did increase the proportion of participants attending screening appointments (OR 1.17, 95% CI 1.03–1.33).

**Conclusions:**

Many reasons given for not participating in trials are not tractable to individual trials. However, modification of the PIL does show potential to modestly improve participation. If further trials could identify similar simple interventions that were beneficial, their net effects could substantially improve trial participation and facilitate recruitment into large trials.

## Background

Large randomized trials are the best method to assess the efficacy and safety of treatments that are likely to have moderate-sized effects [1], but can be costly and time-consuming. It is therefore important to identify methods to streamline the conduct of such trials, including the recruitment of participants [2]. Randomizing large numbers into a trial is frequently a major challenge and numerous interventions to improve recruitment have been tested previously but with varying and typically limited success [3].

Identifying potential participants from routinely collected electronic health records has facilitated recruitment for several large randomized trials in the UK [4–6]. Electronic hospital records are searched to identify patients with appropriate diagnoses and the contact details and relevant diagnostic information of these patients is sent to a central coordinating centre after appropriate ethics and privacy approvals are in place. The coordinating centre then invites (in the name of a local investigator working at the hospital hosting the trial and from where the patients have been identified) potential participants by mail to attend a trial screening clinic at which eligibility is assessed and written informed consent taken.

However, the response rate to such invitations has fallen since its introduction. Between May 1994 and March 1997, 63,603 (49%) of the 130,873 patients invited attended a screening clinic for the Heart Protection Study (HPS, a 2 × 2 factorial trial testing simvastatin 40 mg versus placebo and antioxidant vitamins versus placebo). Later, between July 1998 and August 2001, 34,780 (42%) of the 83,237 patients invited attended a screening clinic for the Study of Effectiveness of Additional Reductions in Cholesterol and Homocysteine (SEARCH) trial (a 2 × 2 factorial trial testing simvastatin 80 mg versus simvastatin 20 mg and folic acid/vitamin B12 versus placebo). Recruitment for the HPS2-THRIVE (Treatment of HDL to Reduce the Incidence of Vascular Events) began in January 2007 and the response rate (after the first 34,000 invitations) had fallen further such that 13% of those invited attended a screening clinic.

This lower-than-expected response rate presented a major operational challenge. Consequently, as part of the ongoing recruitment efforts, several investigations (including two embedded randomized comparisons) were initiated to explore possible reasons for the decline and interventions to mitigate it. It was hypothesized that the provision of the participant information leaflet (PIL) in advance of a study appointment (without the benefit of any verbal discussion of its contents) may put off some potential participants. Our aims were, therefore, to understand the reasons potential participants give for not participating in a trial, and whether attendance at the first study appointment and subsequent entry into the trial could be improved by either (1) providing a summary PIL with the invitation (instead of the full PIL); or (2) using a modified version of the PIL.

## Methods

HPS2-THRIVE was a large international randomized trial of extended release (ER) niacin/laropiprant among patients at high risk of vascular disease. The detailed methods of the HPS2-THRIVE trial are described elsewhere [7]. In brief, HPS2-THRIVE recruited patients > 50 years old with previous cardiovascular disease (prior myocardial infarction, stroke or peripheral arterial disease or other coronary disease if the participant also had diabetes mellitus) as long as there was no clear contraindication to treatment with ER niacin/laropiprant or other major chronic disease that might adversely affect participation. HPS2-THRIVE is registered at ClinicalTrials.gov (NCT00461630). These investigations were conducted as part of the invitation process for UK participants.

### Reasons for potential participants declining invitation

Potential participants were identified from electronic hospital records and sent an invitation letter (on behalf of the local investigator at their local hospital), including the PIL and a reply form (with a freepost envelope). Participants were given a provisional appointment, which they could confirm, change or decline by telephone or decline using the reply form. If they used the reply form to decline, they were asked to record a reason why they did not wish to participate using freetext (which was categorized by a clinician working in the coordinating centre).

### Randomized evaluation of inclusion of summary versus standard PIL

In order to determine whether inclusion of the PIL was reducing the proportion of potential participants accepting the invitation, ethics approval was obtained to randomly allocate at least 10,000 potential participants to receive either:

- The standard invitation package (described above) including the standard PIL (Additional file 1: Appendix 1); or
- A minimal invitation package, which replaced the standard PIL with a brief one-page trial summary (Additional file 1: Appendix 2). In addition, these participants were asked to attend 15 min before their scheduled screening appointment to allow time to read the PIL.

Participants were randomized centrally by the computer programme, which generated the invitations with no stratification or minimisation. The primary outcome for this comparison was the proportion of invited potential participants who were willing and eligible to enter the trial (i.e. entered the pre-randomization run-in phase). Random allocation of at least 10,000 invitations would provide excellent statistical power (> 90% at 2*p* < 0.05) to detect a 2% absolute increase in the proportion entering run-in (i.e. from 10% of those invited to 12%). Differences in proportions were compared using the chi-squared test and odds ratios (with 95% confidence intervals) were calculated. All analyses were based on “intention-to-treat” principles.

### Focus groups on barriers to participation and development of a modified PIL

People, Science and Policy Ltd. (PSP) were engaged to conduct formative research to examine patients’ reactions to the invitation package, to explore reasons for the low response rate and to suggest improvements that might make their peers more willing to participate. This research used focus groups conducted in accordance with the Market Research Society Code of Conduct. PSP staff prepared the topic guides in consultation with Clinical Trial Service Unit (CTSU) staff (which covered the issues raised by the reasons for declining the invitations (see above)) and acted as focus group facilitators to ensure all the topics were discussed sufficiently. Four focus groups were held in June and July 2009, two in London and two in Birmingham. Participants’ characteristics matched the inclusion criteria for the trial (but they were not participating in it); different genders and social classes were represented equally but groups were kept small and relatively homogeneous to encourage active participation.

In addition to the standard invitation package, groups were asked to consider a modified PIL, which was shorter, was in colour and included photographs. Each group meeting lasted 90 min and was audio-recorded. The analysis was conducted by PSP and provided to CTSU; the recommendations were used to inform the development of a new PIL (Additional file 1: Appendix 3).

### Randomized evaluation of a modified PIL versus the standard PIL

Following the focus groups, the PIL was modified in the light of their recommendations. To investigate whether the modified PIL would increase the response rate, ethics approval was obtained to randomly allocate a further 10,000 potential participants to receive either:

- The standard invitation package (described above) including the standard PIL (Additional file 1: Appendix 1); or
- A modified invitation package including the new PIL

The primary outcome (and statistical power) of this comparison were identical to those for the comparison of standard PIL versus a one-page summary of the trial as described above.

## Results

Overall, 228,391 invitations were sent from the coordinating centre to potential participants of whom 24,381 (11%) attended a screening visit.

### Reasons for potential participants declining the invitation

Between January and December 2007, 9427 invitations were mailed. Of these, 4069 (43%) declined the invitation of whom 2554 (63%) did so using the reply form (the remainder declined by phone and no reason was collected for these people). The reasons given are shown in Table 1: 1900 forms included 2362 reasons and 654 people declined without giving a reason.

**Table 1:** Reasons given for not wishing to participate based on 2554 written responses to 9427 invitations

Reason	Number (%) of patients giving reason
Mobility or transportation	679 (27)
Considered their health too poor	376 (15)
Considered themselves ineligible	365 (14)
Considered their health too good	277 (11)
Concern about side-effects	206 (8)
Not interested	174 (7)
Taking too many tablets already	162 (6)
Unable to fast	123 (6)
No reason given	654 (26)

Patients could give more than one reason

### Randomized evaluation of inclusion of the summary versus the standard PIL

Between July and October 2008, 21,156 invitations were randomly allocated to include either the standard PIL or a one-page summary of the trial. There was no difference in the proportion who entered the run-in or who attended their screening visit (Table 2).

**Table 2:** Response to invitation including one-page summary or standard PIL

	One-page summary	Standard PIL
Invitations sent	10,566	10,590
Number (%) attending screening visit	1181 (11.2%)	1122 (10.6%)
Odds ratio (95% CI)	1.06 (0.97–1.16); *p* = 0.17	ref
Number (%) entering run-in	720 (6.8%)	690 (6.5%)
Odds ratio (95% CI)	1.05 (0.94–1.17); *p* = 0.38	ref

### Focus groups

Four major barriers to participation were identified during the focus groups:

1. Changing medication: this was the biggest concern for many members. They were reluctant to change their medications (which they may have been taking for some time), especially if the research team did not understand all the reasons their usual doctor had for selecting a given treatment.
2. Misunderstanding elements of trial design: some were also concerned that the use of a placebo indicated that all current treatment would be stopped and were reluctant to spend 4 years taking a placebo. Some were unaware of what niacin (the main element of the experimental treatment) is and others did not understand why it was being tested in a population who already had disease.
3. Side effects: although members of the focus group acknowledged that all drugs have side-effects, some felt that there was no trade-off with personal benefit or that any damage caused may be permanent. Although many were aware of the TGN1412 episode at Northwick Park where many participants became severely ill [8], most realised that HPS2-THRIVE was a different type of trial and were reassured by the monitoring (although some were concerned that the clinics were run by a nurse rather than a doctor).
4. Timescale: some participants thought 4 years was a long time to commit to and felt they may have to withdraw before the end of the trial (and none mentioned that this might compromise the trial). Others were concerned that stopping the study drug might itself have adverse effects.

Participants of these focus groups were supportive of the method of recruitment, but would expect their own doctor to be aware of the trial before they were contacted. They felt that sharing data (including contact details) within the National Health Service (NHS) is almost expected.

The focus groups then discussed the standard PIL and the modified version. Although almost all participants liked the standard PIL (and felt it looked academic and professional) they felt it was unlikely to be read because the text was too dense. The modified PIL was preferred because it was easier to read because of both its style and layout.

### Randomized evaluation of the modified PIL versus the standard PIL

Between November 2009 and January 2010, 12,164 invitations were randomly allocated to include either the modified or the standard PIL. There was no difference in the proportion who entered run-in, although the proportion who attended screening was significantly greater (Table 3).

**Table 3:** Response to invitation including modified or standard PIL

	Modified PIL	Standard PIL
Invitations sent	6104	6060
Number (%) attending screening visit	580 (9.50%)	499 (8.23%)
Odds ratio (95% CI)	1.17 (1.03–1-.33); *p* = 0.01	ref
Number (%) entering run-in	373 (6.11%)	339 (5.59%)
Odds ratio (95% CI)	1.10 (0.94–1.28); *p* = 0.23	ref

## Discussion

We investigated the reasons why many potential participants declined their invitation to participate in the HPS2-THRIVE randomized trial of ER niacin/laropiprant, following an initial finding that the response rate to the invitation was around 13% compared to around 50% historically in similar trials [4, 5].

A variety of reasons were given for refusing the invitation, among which difficulty with attendance represented about one quarter and was most common. We explored the reasons further in focus groups and by conducting two embedded randomized comparisons investigating whether the inclusion of the PIL with the initial invitation letter was putting people off. We found no evidence that the proportion entering the trial was increased by delaying presentation of the PIL until they attended the clinic (rather than sending it with the invitation). The PIL was modified after focus group discussion and inclusion of this modified PIL did not significantly improve the proportion of potential participants entering the trial, although it did marginally increase the proportion attending their screening appointment.

Our survey of reasons potential participants gave to decline their invitation revealed that many reasons are not modifiable by the trial team. The commonest reason (difficulty accessing the trial clinic) was despite the offer of reasonable travel expenses, suggesting that many patients find it hard to access hospitals (anecdotally, lack of parking is a frequent complaint among hospital attenders). Future trials could be conducted in the community at locations with easier access (or be conducted by mail with no need to attend study clinics [6]) but this would depend on the suitability of the intervention. There was some overlap between the reasons given by participants invited into HPS2-THRIVE and those people selected for the focus groups. One such area was concern about side-effects. Guidelines for writing PILs requires investigators to provide information about risks of physical or psychological harm and to the individual’s confidentiality [9]. Whilst the guidance recognises that conveying such information is challenging, it is now generally recommended that the PIL is provided in advance of informed consent being taken. A consequence of this is that research staff are not available to discuss the possible balance of benefits and side-effects of the intervention, possibly resulting in potential participants being deterred from participation.

It is possible that recommendations by bodies responsible for trial governance and regulation have had the unintended consequence of making trials less appealing to potential participants. However, we conducted two embedded randomized comparisons focussing on the PIL: we found no evidence that delaying provision of the standard PIL until 15 min prior to the screening visit (compared to providing it with the initial mailed invitation) led to more participants attending the screening or agreeing to enter the trial. This is concordant with a previous smaller study that compared the provision of either a long or short information leaflet about a research study to parents of immature infants admitted to neonatal intensive care [10]. There was no difference in enrolment rate between the two arms (although only 41 parents participated); also of interest, is the finding that recipients of the longer (more detailed) information actually had less understanding of the trial procedures (assessed by questionnaire) than those provided the shorter information. Similarly, an embedded comparison in a trial of computerized cognitive behavioural therapy in primary care also found no difference in trial participation between potential participants sent a short PIL (63/1165 (5.4%)) or a standard-length PIL (59/1165 (5.1%)) [11].

We also tested whether a different presentation of the PIL (following discussion at the focus groups) would improve participation. Although this did not significantly increase the proportion of potential participants entering the trial, it did significantly increase the proportion who attended a screening visit by about one sixth (Table 3). The consistency between the odds ratios of these two outcomes suggests that - had the comparison been larger (than the 12,000 invitations included) - the effect on entry into the trial may have been significant. Although moderate in size, the improvement in absolute terms of 0.5% would have reduced the number of invitations required to screen the 16,000 UK participants by about 5000 and shortened the recruitment phase by several months, which would both be worthwhile outcomes, not least because of the reduced workload demanded at the coordinating centre to generate this number of invitations. Indeed, if other interventions with similar moderate effects were identified they could in total lead to significant savings in terms of financial costs and time required to recruit into similar large randomized trials. Conversely, if modification of the PIL increases attendance at screening clinics but has no effect on the proportion entering run-in then this increase in workload would be undesirable.

Our analysis of the reasons potential participants gave for not accepting their invitation did not include the reasons given by those who chose to respond by telephone, who may differ from those who chose to decline by post. However, the focus groups provided a more systematic assessment of potential participants’ concerns about entering a large randomized trial. Although many of the reasons given are not tractable within individual trials, improving public understanding of trials and their role in health care could improve the willingness of people to enter trials. The randomized comparisons here focussed on one aspect of the PIL each (either the timing of its presentation or its style). Further similar work could be conducted to assess whether other modifications to the PIL (such as using electronic, animated or video versions) could improve recruitment further, and on other aspects of trial recruitment, since most interventions tested to date have had no or very limited impact [3].

In summary, potential participants give several reasons why they do not wish to participate in trials, including concern about side-effects presented in the PIL. However, delaying presentation of the full PIL until a member of the research team was available to discuss it did not improve trial participation, but modifying the style of the PIL to make it more accessible did appear to improve potential participants’ willingness to enter a trial.

## Supplementary information

**Additional file 1: **Coordinating centre and collaborators. **Table S1.** Baseline characteristics of participants included in randomized comparisons of (A) summary PIL versus standard PIL and (B) modified PIL versus standard PIL. **Appendix 1.** Original participant information leaflet. **Appendix 2.** Invitation letter with one-page trial summary. **Appendix 3.** Modified participant information leaflet.

## Data Availability

Data sharing will be considered in line with the Nuffield Department of Population Health, University of Oxford, Data Access and Sharing Policy available at https://www.ndph.ox.ac.uk/about/data-access-policy.
