# Supplementary material for: Investigating modifications to participant information materials to improve recruitment into a large randomized trial
Source: Trials. 2019 Dec 5;20:681. doi: 10.1186/s13063-019-3779-4 (PMC6896768; doi:10.1186/s13063-019-3779-4)
Supplement: Supplementary file 1 — Additional file 1: Coordinating centre and collaborators. Table S1. Baseline characteristics of participants included in randomized comparisons of (A) summary PIL versus standard PIL and (B) modified PIL versus standard PIL. Appendix 1. Original participant information leaflet. Appendix 2. Invitation letter with one-page trial summary. Appendix 3. Modified participant information leaflet. [file 13063_2019_3779_MOESM1_ESM.pdf]

Investigating strategies to improve recruitment into a large randomized trial

SUPPLEMENTARY APPENDIX

Table of Contents

Coordinating centre and collaborators

Supplementary Table 1: Baseline characteristics of participants included in randomized comparisons of A: summary PIL versus standard PIL; and B: modified PIL versus standard PIL.

Appendix 1: Original Participant Information Leaflet

Appendix 2: Invitation letter with one-page trial summary

Appendix 3: Modified Participant Information Leaflet

Coordinating centre and collaborators

Central Coordinating Office, UK Regional Coordinating Centre and China Group (Clinical Trial Service Unit, University of Oxford): *Administration and support* C Bray, R Dayanandan, E Wincott (coordinators), P Achiri, C Anderson, T Bahu, S Barry, I Boller, A Brewer, A Brewster, R Brown, A Burke, E Butler, Y Chen, M Corbett, J Crowther, S Danesh-Pour, R Davis, H Demarest, K Frederick, A Fortun, T Gordon, M Gray, T Grimsey, N Guy, C Harwood, J Heineman, K Holt, C Hope, S Howard, K Jayne, M King, S Knight, H Lang, A Lewis, O Machin, Z Madgwick, C Marsden, M Matthewson, S Michell, J Miller, K Murphy, E Nicholson, J Nie, J Nolan, M Nunn, A Panicker, S Pickworth, Y Qiao, A Radley, L Rowley, C Sabine, J Sayer, A Taylor, K Taylor, H Thorne, M Wickman, K Yu; *Clinical support and adjudication* J Armitage, T Aung, C Baigent, L Bowman, R Bulbulia, F Chen, Z Chen, R Clarke, T Dasgupta, S Dunachie, R Haynes, W Herrington, L Hirt, M Landray, D Lewis, W Majoni, C Murray, K Naessens, K Rahimi, C Reith, E Sammons, M Taylor-Clarke, J Tomson; *Computing coordinators* A Baxter, M Lay, K Wallendszus; *Statistics and computing* R Ait Sadi, K Anderson, I Barton, C Berry, C Bird, J Booth, M Bowes, Y Bu, R Clack, P Cleverley, G Coates, J Cox, M Craig, J Crisp, G Cui; P Dalton, C Daniels, A Economou, A Field, S Gilbert, H Gonzalez, R Goodenough, N Goodwin, P Harding, JC Hopewell, R Thomas-Kurien, S Lambern, G Lancaster, A Maskill, P Morris, Y Mostefai, S Mulay, A Munday, A Murawska, A Offer, S Parish, N Prajapati, R Reid, S Shams, S Syed, M Turakani, E Valdes-Marquez, A Young, AC Young, W Zhu; *CTSU Wolfson Laboratory* S Clark, K Emmens, M Hill, K Kourellias, M Radley, J Wintour (coordinators); M Allworth, A-M Beneat, L Boggs, T Chavagnon, A Gordon, J Gordon, C Guest, C Hickman, J Hill, R Hrusecka, N Luker, N Plunkett, L Sansom, R Shellard, J Taylor, P Taylor, J Wheeler, T Williams, M Yeung; *Nurse monitors and trainers* C Knott (coordinator) S Beebe, K Bowsher-Brown, L Bromhall (deceased), J James, A Lawson, H Lochhead; **China Regional Coordinating Centre, Beijing:** *Clinical support and adjudication:* F Feng, Y Gao, J Li, J Liu, X Pang, D Zhang, H Zhang; *Administration and support:* H Dai, F Feng, J Li, F Liu, L Ma, Y Sun, R Tang, M Xie, X Wei, X Yan, M Yang, H Zhong, L Zhang, S Zhang, Y Zhang; *Monitors and Trainers:* L Chen, F Feng, Y Gao, W Hundei, J Li, L Li, J Liu, X Yan, A Zhang, D Zhang, H Zhang;

Laboratory: L Hou, X Li, Z Liu, Q Song, S Zhang; Financial: X Yao, Y Yao, Y Yu; *IT Support:* H Dai, D Deng, X Wei, L Xu, J Zhang; **Scandinavia Regional Coordination:** *Coordinators* K Arnesson, K Staerkebye, G van Leijenhorst, G Moen; *Monitoring training and support Finland:* A Tiiska, T Vaine, S Roine, S Eronen, L Huponen, T Häikö, H Salonen; *Research Director:* P Koskinen, *Sweden:* L Bergvall, J Levin, I Ek, D Vlaheli, L Hedlöf, C Olsson, T Rasmusson, L Hugo, P Cöster; *Norway:* V Larsen, I E Thorsby, N Eriksen, V Bjorhovde, K Jordheim, I L Östman; *Denmark:* T Johansen, S Mosegaard, H Diget, M Snejbjerg, O Moll. **China Collaborators** *Department of Cardiology, The First Hospital of Jilin University, Changchun:* Y Zheng, W Zhao, D Chen, S Zhou, H Sun; *Department of Endocrinology, The First Hospital of Jilin University, Changchun:* Q Liu, X Gang, J Ning, W Guo, L Gao, M Li; *China-Japan Union Hospital of Jinlin University, Changchun:* P Yang, M He, M Ding, Z Feng; *The General Hospital of FAW, Changchun:* H Pan, X Wang, S Zhang, Y Liu, H Liu; *Suzhou Kowloon Hospital:* F Liu, T Zhang, B Shao, J Yu; *The 306th Hospital Of P.L.A., Beijing:* Z Xu, X Li, L Shi, Z Wang, Z Li; *Peking University People's Hospital, Beijing:* N Sun, H Wang, J Wang, F Yang; *Peking University Third Hospital, Beijing:* W Gao, B Chen, X Wang, Z Li, H Wang; *Peking Union Medical College Hospital, Beijing:* S Zhang, J Yan, Z Liu, J Yang, L Li, W Chen, H Bai, S Wang; *Department of Heart Surgery, Fuwai Hospital, Beijing:* Z Zheng, X Wang, X Chen, Y Zhao, X Pan, C Zhang; *Department of Cardiology, Fuwai Hospital, Beijing:* Y Yang, J Song, L Li, D Song; *Beijing Chaoyang Hospital, Capital Medical University:* X Yang, X Lin, F Zhang, M Chen, X Gao, J Li; *Shandong Weifang People's Hospital:* Y Zhang, L Zhao, H Song, H Li; *Department of Cardiology, Tianjin Medical University General Hospital:* Z Wang, Y Sun, W Zhang, Y Li, Y Xu; *Department of Neurology, Tianjin Medical University General Hospital:* Y Cheng, M Zou, J Han, X Tian; *The Second Hospital Of Tianjin Medical University:* X Li, Y Du, W Lu, W Qiu, Y Wang; *Department of Cardiology, Tianjin Union Medicine Centre:* K Liu, S Li, Y Liu, R Wang, C Lyu; *Department of Neurology, Tianjin Union Medicine Centre:* C Zhang, G Chen, C Ma, H Ma, R Zhang; *Department of Cardiology, Tianjin Third Central Hospital:* H Wang, J Luo, S Pi, F Feng; *Department of Neurology, Tianjin Third Central Hospital:* Z Zhang, X Zeng, J Zhang;

Tianjin Fourth Central Hospital: H Zhang, Y Li, Y Liu, Z Chen; *Tianjin Chest Hospital:* X Guo, J Zhao, S Han, Y Wang; *The General Hospital Of AISCO, Anshan:* W Zhao, X Liu, L Li, L Liu, D Jiao; *Shuangshan Hospital Of Anshan:* R Xiao, R Wu, X Li, Y Lu; *Tiexi Hospital of Anshan:* B Liu, Z Wang, X Bian, Y Shao; *Department of Cardiology, The People's Hospital Of Liaoning Province, Shenyang:* Z Li, R Cui, Y Liu, D Li, G Dai; *Department of Neurology, The People's Hospital Of Liaoning Province, Shenyang:* X Chen, X Li, Y Wei, Y Lu, Q He, L Zheng; *The Fourth People's Hospital Of Shenyang:* Y Li, X Guan, X Zhou, W Jiang; *Department of Cardiology, The First Hospital Of China Medical University, Shenyang:* G Qi, C Wu, Z Jia, X Li, P Li; *Department of Neurology, The First Hospital Of China Medical University, Shenyang:* C Zhang, Q Li, X Zhang, X Li, C Xu; *Shengjing Hospital of China Medical University, Shenyang:* S Ma, Y Sun, Y Fu, Y Chen, J Shi, H Li, M Zhao, D Zou, Z Zhao; *Department of Cardiology, The First People's Hospital Of Shenyang:* F Feng, J Xu, W Xing, J Wang; *Department of Neurology, The First People's Hospital Of Shenyang:* G Tian, J Zhou, L Li; *The Fourth Affiliated Hospital Of China Medical University, Shenyang:* Y Jin, X Zhou, Y Lin, L Ye; *Liaoning Shenyang Sujiatun District Central Hospital:* H Che, W Feng, D Li, J Guo; *The Fifth People's Hospital Of Shenyang:* Q Diao, Z Liu, H Wang, L Yuan; *Department of Cardiology, Baogang Hospital, Baotou:* Z Ge, L Wei, Q Liu, L Gong; *Department of Neurology, Baogang Hospital, Baotou:* D Wang, H Wang, L Li, J Hou; *The Second Affiliated Hospital of Baotou Medical College:* G Sun, G Wang, G Huang, Y Ding; *The First Affiliated Hospital of Baotou Medical College:* C Wang, J Zhang, D Liu, Q Yang, J Chen; *Inner Mongolia Autonomous Region Hospital, Hohhot:* Y Han, W Lu, Y Zhang, S Dong; *The Affiliated People's Hospital of Inner Mongolia Medical College, Hohhot:* J Liu, L Chen, L Geng; *Department of Cardiology, Tongji Hospital, Tongji Medical College, Wuhan:* D Wang, J Yan, C Xu, C Chen; *Department of Neurology, Tongji Hospital, Tongji Medical College, Wuhan:* W Wang, X Luo, S Xu, D Tian, X Meng; *The First Hospital of Wuhan:* G Chen, L Luo, X Pan, X Rao; *Department of Cardiology, Wuhan Puai Hospital:* Y Gu, L Li, Z Cheng, X Liu; *Department of Neurology, Wuhan Puai Hospital:* J Wang, X Zeng, Z Luo, Q Yan; *Central Hospital of Wuhan:* S Zhao, L Chen, J Chen, L Guo, N Li, A Fu; *Wuhan Asia Heart Hospital:*

X Su, S Zhao, X Zhai, W Wu, J Zheng; *Department of Cardiology, The First Affiliated Hospital of Zhengzhou University:* Z Huang, X Fu, H Yao, X Zhang; *Department of Neurology, The First Affiliated Hospital of Zhengzhou University:* B Zhang, Y Liu, Z Fu, X Ma, H Wei; *Department of Cardiology, The Second Affiliated Hospital of Zhengzhou University:* Y Zhao, Q Zhang, J Dong, H Li; *Department of Neurology, The Second Affiliated Hospital of Zhengzhou University:* J Lou, X Yang, C Cui, J Su, X Zhang; *The Central Hospital of Zhengzhou:* L Zhang, H Sun, H Li, R Wang; *Department of Cardiology, The Affiliated Hospital of Xuzhou Medical College:* D Li, T Xu, W Wu, H Zhu, X Ma; *Department of Neurology, The Affiliated Hospital of Xuzhou Medical College:* X Shen, Z Zhang, J Lu; *Department of Cardiology, Xuzhou Central Hospital:* Q Fu, A Zhao, L Wang, J Li; *Department of Neurology, The Central Hospital of Xuzhou:* Z Yu, X Zhang, C Yang, Y Ling; *Department of Cardiology, Xuzhou No 1 People's Hospital:* H Zhang, L Li, Y Shang, M Hu; *Department of Neurology, Xuzhou No.1 People's Hospital:* L Zhou, D Kong, N Wang, H Shang; *Department of Cardiology, The General Hospital Of Xuzhou Mining Group:* W Wu, J Feng, L Li, X Yang; *Department of Neurology, The General Hospital Of Xuzhou Mining Group:* L Rong, X Wei, A Gong, J Dong; *The Third Peoples Hospital of Xuzhou:* L Wang, X Tang, C Zong, C Zhao; *Department of Emergency, The Affiliated Hospital of Medical College Qingdao University:* C Zhou, Y Wang, M Guo, X Yang; *Department of Neurology, The Affiliated Hospital of Medical College Qingdao University:* H Pei, H Li, D Han; *Department of Emergency, Qingdao Municipal Hospital:* F Zhang, D Hou, Q Zhang, Y Li, Z Sun, L Wang; *Department of Cardiology, Qingdao Municipal Hospital:* X Wang, Y Wang, Y Yao; *Chinese Academy of Medical Science, Qingdao Fuwai:* Y Yang, G Wu, Y Chang, J Mao; *Department of Cardiology, The First Clinical College of Harbin Medical University:* W Li, X Zhang, B Liu, M He, J Jing, J Feng, Y Zhang, Y Bao; *Department of Neurology, The First Clinical College of Harbin Medical University:* L Zhang, Y Sun, B Liu, S Zhou, X Wang, Y Zhang; *Department of Cardiology, The Second Affiliated Hospital of Harbin Medical University:* B Yu, Y Luan, W Cao, Y Wang, L Diao; *Department of Neurology, The Second Hospital of Harbin Medical University:* W Wang, D Yang, Y Zhu, L Liu, X Meng; **UK Collaborators** *Aberdeen Royal:* J

Webster, H Clark, J Henderson, V Herd, P McDonald; *Addenbrooke's, Cambridge:* M Brown, F Mir, S Blackwood, S Hood, G Carr; *Barnsley:* B Saeed, W Khan, M Tahir, K Elliott, M Reid; *Bedford:* I Cooper, A Gallivan; *Birmingham City:* A Basu, B Gammon, S Poxon; *Birmingham Heartlands:* A Jones, L Andrews, E Burke, M Drew, C Jewkes; *Blackpool Victoria Hospital:* D Roberts, L Hutt, S Pickervance, L Radford, M Jones; *Bradford Royal:* S Lindsay, C Atkinson, K Rees, A Wilson, P Brown; *Bristol Royal:* G Bayly, J Chambers; *Cheltenham General:* R Bulbulia, K Poskitt, P Hilltout, S Smith, J Rawlings; *Colwyn Bay:* C Bellamy, E Horner; *Conquest Hastings:* R Wray, H McIntyre, H Pepper, J Sinclair; *Worcestershire Royal Hospital, Worcester and Corbett Medical Practice, Droitwich Spa:* A Munro, J Trevelyan, V Wilkie, J Colley, A Doughty; *Cumberland:* H Robson, K Willmer, T Joseph, P Davies, M Cowley, A Graham, C Hagon, R Jolly, N Kelsall, U Poultney, E Warwick; *Darlington Memorial:* J Murphy, G Brennan, S Stubbing, M Boon; *Derriford:* J Fulton, C Davison, J Simmonds, R Squire, C West, S Pedlar; *Dewsbury:* H Chidambara, G Bateman, M Khalifa; *Dorset County:* T Edwards, B Anderson, S Breakspear, L O'Shea, P Jeffreys; *Eastbourne:* G Lloyd, N Patel, H Bailey, S Carter, J Large; *Edinburgh Royal:* D Newby, H Cuthbert, L Flint, H Nailon, J Robertson; *Frenchay, Bristol:* M Papouchado, G Andrews, N Manning; *Glenfield, Leicester:* I Hudson, P DeSouza, S Mellor, K Henson; *Guy's and St Thomas:* J Chambers, A Wierzbicki, D Grant, D Parkin; *Harrogate:* P Hammond, C Bennett, A Wray; *Hillingdon:* R Hillson, M Edwards, N Mahabir, Y MacDonald; *Hospital of St Cross, Rugby:* P O'Hare, B De Burca, J Jones, J Wykes; *Huddersfield Royal:* H Griffiths, A Burrows, D Appleyard, A Wilson, M Habron, A Kudarenko; *King's Mill, Sutton-in-Ashfield:* R Lloyd-Mostyn, I Idris, P Bushby, S Dale, S Hardingham, S Thomas, V Godfrey; *Leighton, Crewe:* S Mallya, A Heald, J Maiden, J Rhodes, S Tapscott, K Dillon; *Lister, Stevenage:* M Lynch, A Viljoen, W Burog, M Dhaliwal; *Luton and Dunstable:* C Travill, S Gent, R Hering, A Wilkinson, D Shah; *Macclesfield:* R Egdell, E Rickards, A Taylor Bennett; *Manor, Walsall:* P Giles, A Hartland, A Isaew, E Walton, K Parry, L Hawkins; *Morriston, Swansea:* P Thomas, C Weston, C Fagan, E Hopkins, C Jones, S Davies, T Ghuman; *Musgrove Park, Taunton:* R Andrews, N Salter; *Neath Port Talbot:* C Jones, A Owen, P Thomas, S Meredith, C Thomas, E Alford; *Ninewells,*

Dundee: T Pringle (deceased), S Pringle, H Waldie, S Hutcheon, G McNeill, K Smith; *Norfolk and Norwich University Hospital:* E Roberts, A Ryding, I Gallagher, T Harris, M Ilsley; *North Manchester General:* J Swan, D McSorland, C O'Neill; *North Tyneside General:* R Curless, C Doig, P McKenna, M Armstrong; *Northampton General:* J O'Donnell, P Davey, G Nayani, L James, K Smith, E Tanqueray, C Hemmington, P Mackenzie, S Russell; *Northwick Park:* R Senior, J Evans, A Sinton, B Sparkes; *Peterborough City:* D Rowlands, J Porter, T Ahmed, S Hamill, S James, M Mortimer, T Peachey; *Pinderfields, Wakefield:* C White, H Chidambara, G Bateman, M Khalifa, J Massarella; *Poole:* A McLeod, B Howarth, F Keene, K Knops, P Jeffery; *Princess Royal, Telford:* N Capps, D Donaldson, L Tonks*: Queen Elizabeth, Birmingham:* J Townend, C Aston, E Dwenger, T Mistry, C Crook; *Queen's Hospital, Burton-upon-Trent:* T Reynolds, M Jones, J Reynolds, K Pennlington; *Queen's Medical Centre, Nottingham:* P Mansell, T Gazis, B Mendis, A Selby, K Ward, J Wallis; *Richard Doll Building, Oxford:* J Armitage, M Landray, H Watkins, A Alexander, S Beebe, L Bromhall (deceased), K Bowsher-Brown, H Cowan, A Isaew, J James, A Lawson, H Lochhead, V Toghill, L Wright, D Jackson, A Taylor; *Rotherham General:* R Muthusamy, A Jahangir, C Weston, V Darlow; *Royal Berkshire:* W Orr, J Foxton, J King; *Royal Blackburn:* S Ramtoola, J McDonald, Y Grimes, K Rutherford, F Ibrahim; *Royal Bolton:* A Hutchesson, K Morris; *Royal Bournemouth:* A Rozkovec, S Orr, P Ryder, C Thomas, J Walker, A Sinclair-Brackstone; *Royal Cornwall:* S Fleming, M Parrett, L Quinn, B Howes, P Ivanovic, A James, R Soper*; Royal Derby:* G Tan, F Game, A Boyal, T Gibson, J Maiden, V Mountford, J Reynolds, S Yates; *Royal Devon and Exeter:* K MacLeod (deceased), M James, S Estcourt, E Green, S Henson; *Royal Hallamshire, Sheffield:* T Gray, S Song, K Birchall, A Creaser-Smith, E Goodwin, C Morgan, A Nazir, M Stringer, J Tandy, C Dakin, J Williamson, H Dunn, L Strong, S Roney, T Standering; *Royal Surrey County:* T Chua, C Livingstone, C Blythe, M Payne, A Selby; *Royal Sussex County:* D Hildick-Smith, E Joyce, N Skipper, G Bassett; *Royal United, Bath:* J Reckless, G Andrews, J Howard, C Shute, S Pope; *Royal Victoria, Belfast:* P Nicholls, I Young, N Brown, S Loughridge, M Blaney, L Bradford; *Royal Victoria, Newcastle:* I U Haq, C Albers; *Russells Hall:* M Labib, M Allen, L Higginson, A Zhao, C Ford, L Wright; *St Helens,*

Liverpool: J Ball, K Hardy, L McCulloch, M Sheridan, A Wilson, D Mair; *St Helier:* J Barron, H Wilcox, M Lapsley, B Bradford, M McIntosh; *St Marys, Portsmouth:* P Kalra, A Siva, D Barnes, S Golledge, K Hudson, A Suttling, M Ambler; *St Richard's, Chichester:* Y Wong, C Murphy, C Reid, S Moore, S Stearn; *Salford Royal:* A Fitchet, P Kingston, K Morris; *Sandwell General:* E Hughes, B Gammon, S Poxon, C Verow, N McCarthy, R Kumar; *Scunthorpe General:* J John, S Chattopadhyay, D Briggs, C Gray, D Bruce; *Southampton General:* C Shearman, C Sibley, E Hayward, D Tyler; *Stafford :* K Evans, J Bellaby, C Wood; *Stepping Hill, Stockport:* P Lewis, A Brown, H Cochrane, J Curtis, S Scanlon; *Sunderland Royal:* S Junejo, M Farrer, L Cowell; *Torbay:* C Carey, J Sutton; *University Hospital, Coventry:* M Been, B De Burca, J Jones, J Wykes; *University Hospital of North Staffordshire:* J Creamer, K Castro, J Machin, E Sellars; *University Hospital of Wales:* I McDowell, P Groves, L Davies, H Dyer, C Dennison; *Victoria Hospital, Kirkcaldy:* M Francis, V Bryson; *Walton Centre:* R White, P Enevoldson, A Amadi, K O'Hanlon, S Saminaden, L Wyatt, A Atkins; *Watford General:* M Clements, F Lukwago, K Markwell, E Walker, E Atkins; *West Cumberland, Whitehaven:* K Willmer, O Orugun, D Koziara R Jolly, U Poultney; *Whipps Cross University Hospital:* F Lie, J Hogan, V Conteh, M Montemayor, N Mooneyan, V Ramasamy, H Sizeni, D Hart; *Worthing:* M Signy, R Chilton, R Gomez Marcos, G Peters; *Wycombe:* S Price, N Mahabir; *Wythenshawe:* S Ray, D Daniel, J Dean-Oshodi; *Yeovil District:* G Brigden, G Chung, C Buckley, C Vickers. **Scandinavian Collaborators: Denmark** *National coordinator:* K Egstrup *Collaborators: Sygehus Fyn, Svendborg:* K Egstrup, T Sønder, A Osmanagic, T Brøcher Christophersen, T Erstad, D E Høfsten, M Løgstrup, K Vinther, M Lundegaard, A Due, M Hansen; *Regionshospitalet, Randers:* J Bertelsen, H Rickers, T Jensen, B Winter Friis, M Morsing, M Rasmussen; *Sydvestjysk Sygehus, Esbjerg:* K Thomsen, J Hummelshoej, A Nygaard, L Knudsen, T Nielsen, L Tanggaard; *Roskilde Sygehus, Roskilde:* G Jensen, T Melchior, S Heinsvig, I Larsen, V Perrit-Gentil; *Hvidovre Hospital, Hvidovre:* S Rasmussen, N Skov, K Meier; *Glostrup Amtssygehus, Glostrup:* J Nielsen, J Abdulla, J Berning, J Jensen, J Jeppesen, M Seibæk, A-L Jorgensen, M Bulow; *Gentofte Amstssygehus:* Hellerup*:* N E Bruun, C Larsen, R Vedby-

Rasmussen, L Fahlström, K Ladegaard- Rasmussen, H Bjørnager, A Holdgaard; *Kolding Sygehus, Kolding:* A Dorph Petersen, O Nyvad, J Meinertz Byg, P Wiggers, L Schmidt-Thomsen, L Holm-Pedersen, B Felthaus, J Mørkeberg; *Regionshospitalet, Viborg:* I C Klausen, E Steen-Nielsen, B Hedegaard, E-M Dalbjerg; *Holbæk Sygehus, Holbæk:* N Roseva-Nielsen, D Hansen, H Madsen, K Kristensen, H Ibsen, K Schultz, B Fredenhardt, J I Nielsen, L Petersen, T Bang-Hansen, L Jensen, C Justesen, M Storm Larsen, T Veng-Olsen; *Herning Sygehus, Herning:* J U Brorholt-Petersen, M Andersen, V Lynggaard, S Vester, S Hansen, B Larsen, L Madsen; *Herlev Sygehus, Herlev:* K Skagen, U Schmidt, T Poulsen, H Christensen, J Hansen, J M Appel, A Therkelsen; *Gentofte Hospital 2, Hellerup:* C Pedersen, S Andersen, D-M Bretler, T Schram, J Madsen, G Von Jessen, M Nielsen, K Serup-Hansen; *Rigshospitalet, Copenhagen:* P Clemmensen, P Grande, B Thomsen, K Graversen, L Hedegaard, H Mirchandani; *Helsingoer Sygehus, Helsingoer:* N Ralfkiaer, P Lunn, S Nielsen, H Gottschalck; *Haderslev Sygehus, Haderslev:* L Lemming, F Davidsen, M Hansen, C Markussen; *Aalborg Sygehus, Aalborg:* E B Schmidt, H Vadmann, T Andersen, T Madsen, M Rene Nielsen, J Stegger, M Storgaard, L Kobbelgaard, G Mikkelsen, A Andreasen, *Kolding Sygehus, Kolding:* J Jepsen, J Gesla, L Holm-Perdersen, B Kaspersen, B Eskesen, M Loekke; *Kollesterollaboratoriet, Gentofte:* S Stender, M Andersen, H Jonsson, T Brink-Kjær, M Bülow, A-L Jørgensen, N Petersen, A M Raabyemagle, L Koch; *Slagelse Sygehus, Slagelse:* J Lomholdt, M Lessing, K Niemann, H Mollerup; *Næstved Sygehus, Næstved:* J Thorsen, P Elin, B Jensen. **Finland** *National coordinator:* A Kesaniemi *Collaborators*: *Oulun yliopistollinen sairaala, Oulu:* A Kesaniemi, O Ukkola, E Eloranta, L Ukkola, P Ojala, L Mannermaa; *Helsingin yliopisto, Helsinki:* J Tuomilehto, C Sarti, P Sintonen, R Lehtisalo, M Mähönen, E Lahdensuo; *Vantaa Tutkimuskeskus, Vantaa:* K Humaloja, M Minkkinen, U Nopola-Humaloja, K Jarvinen, K Azezian, J Vaerma; *Kuopion liikuntalaaketieteen tutkimuslaitos, Kuopio:* T Lakka, G Smirnov, H Tuomilehto, N Musakka , M Merikoski; *Tampereen tutkimuskeskus, Tampere:* A Hiukka, A Putila, J Hietala; *Mediwest Seinajoki, Seinäjoki:* T Haapaniemi, S Pihlaja, L Viro, S Viisteensaari*: CorusFit Oy, Jyvaskyla:* M Perhonen, M Valtonen, M Varakas, M Moilanen, A Kivisari, M Kuparinen;

Kuusankosken terveysasema, Kuusankoski: ML Tuominen, A Haaraoja; *Etelä-Karjalan keskussairaala, Lappeenranta:* E Hussi, S Valpas, A Keskitalo; *Kansanterveystieteenlaitos, Helsinki:* J Tuomilehto, C Sarti, R Lehtisalo, M Mähönen, P Sintonen, E Lahdensuo; **Norway** *National coordinator:* D Atar *Collaborators: Oslo universitetssykehus, Oslo:* D Atar, P Vanberg, K Jahnsen, S Kjellesvik; *Ulleval universitetssykehus, Oslo*; T Pedersen, T O Klemsdal, R Kleve, L Flakk; *Ostlandske Hjertesenter, Moss:* P-A Sirnes, V Nilsen, A-K Hammersmark; *Skedsmo Medisinske Senter, Skedsmokorset:* K Risberg, H Hansen, M Berg, G Ronaas; *Medi3 Innlandet , Hamar:* H-O Hoivik, B Furuseth, S Nystuen, S Paulsen Sundeng; *Sykehuset Innlandet, Hamar:* K Andersen, H Kydland, E Søby, T Larsen, H Slettmoen, E K Væringstad; *Sykehuset Innlandet, Lillehammer:* M Grundtvig, L E Erdal, G Szacinski, M German; *Legegruppen Grandgarden, Arendal:* B Bratland, M-B Hornnes, G Sporaland, F Johnsen, O Starheim, G Mouland, J Vikingsdal, A L Torgersen; *Stavanger Helseforskning, Stavanger:* P Munk, T Jonassen, J Cooper, N Butt, T Aarsland, J M Nilsen, A G Larsen, S Moen; *Haugesund Hjertepraksis, Haugesund:* R Rød, R M Stodle, *Haukeland universitetssykehus, Bergen:* C Berge, O Nygaard, N Tuseth, G Svingen, H Schartum-Hansen, J E Nordrehaug, I A Oppedal, B Gjellefall, S Thorsen, J Dyngeland; *Fana Hjertesenter, Nesttun:* P K Ronnevik, T Ronnevik, A Bråten; *Spesialistsenteret, Straume:* B K Oie, A-M Thunestveit; *Trondheim Hjertesenter, Trondheim:* G Skjelvan, S Strand; *Hjerteklinikken, Harstad:* K A Hofsoy, A L Reiss, E Bruun; *Helse sunnmøre, Ålesund:* J Å Vegsundsvåg, E Holte, M Skjold, H Nes; *Sykehuset Innlandet, Kongsvinger:* J Sparby, E Werenskiold, A Faret,; *Heimdal Helsehus Legesenter, Heimdal:* I M Jørum, B Korssjøen, O Gaupen, L Løvaas, R Myran, P Schrøder, A-S Jørum, H Berdal, B Myklebust; *Sinsenklinikken, Oslo:* O Karstad, L Hektoen, M Andresen, J Kristensen, E Karstad; *Helse Førde, Førde:* T Vingsnes, Å Johnsen, A Bjørkås, I Vassbotten; *Lund legesenter, Kristiansand:* S Ø Thomassen, S Bø, L Hæstad, C Ingebretsen, M Marcussen; **Sweden** *National coordinator:* L Jonasson *Collaborators: Universitetssjukhuset, Linköping:* L Jonasson, B Hedbäck, M Börjesson, A Raschperger, M Hedbäck; *Höglandssjukhuset, Eksjö:* P Cherfan, M Norman, Y Pantzar; *Centrallasarettet, Växjö:* P Vasko, M Yngvesson, S

Johansson, I Uggeldahl, M Sjöö-Boqvist; *Närvårdskliniken, Finspång:* A-M Frödenberg, A Rudenäs, A-L Neumann; *Rättviks Hälsan, Rättvik:* H Larnefeldt, M Björkman-Larnefeldt, V-A Lindvall; *Ljungby lasarett, Ljungby:* A Andersson, K-A Svensson, C Johnsson, C Nilsson, M Millborg, A-M Ohlin, A Solberg; *Central Sjukhuset, Kristianstad:* I Lager, I Torstensson, A Knutsson, K Hårsmar; *Capio Citykliniken, Lund:* C-J Lindholm, J Thulin, E Assarsson, M Broberg, H Trege; *Göingenkliniken, Hässleholm:* G Stehn, C Lindgren, M Åkerberg, M Ulfvik; *Alingsås lasarett, Alingsås:* H Nordström, J Sörbo, L Back, V Carter; *Hjärthuset AB, Varberg:* T Carlsson, C Nemeczek, A Nemeczek, A Sterner-Bengtsson; *Två medicinare, Järfälla:* P-Å Lagerbäck, B-O Tengmark, B Bjurman, L Eng-Sjoberg; *Capio City Klinik 2, Lund*; C-J Lindholm, U-B Ericsson, J Thulin, K Hansson, E Assarsson, M Broberg; *Lindesbergs lasarett, Lindesberg:* A-S Eriksson, A Stenberg, T Fraser, D Ticic, A Gunnarsson, H Lindrooth, K Molander; *PRIM site, Örebro:* E Ackefelt-Frick, M Berglund, R Ellervik-Forest, P Fardelin, F Freire, E Norgren-Holst, K Eriksson, U Larsson, H Samuelsson, L Garö, J Kjellberg-Eriksson, A Eklöf, E Carlsson, L Irvhage, P Jaensson, E Melander, I Nordin-Olsson, B Strandell, H Udd, A Krantz; *Universitets Sjukhuset, Malmö:* A Gottsäter, A Alhadad, M Eriksson, B Palmqvist; *Bergslagssjukhuset, Fagersta:* E Hammarström, P Nicol, S Holmström, I-B Svennberg, I Ojakangas, I Andersson; *Danderyds Sjukhus, Danderyd:* K Knudsen-Malmqvist, N Östlund-Papadogeorgos, H Tjerneld, M Nilsson, C Andersson, M Asperen; *City Heart, Stockholm:* C Höglund, L Hjelmaeus, R Zlatewa, I McLain, E Lindemann, B Rangman; *Skellefteå lasarett, Skellefteå:* J-H Jansson, B Norrfors, L-M Lundmark, M Olofsson; *City Site, Stockholm:* B-O Tengmark, S Gunvarsdotter, C Andersson, M Dewerand; *Silentzvägens Vårdcentral, Uddevalla:* G Dahlen, B Polhem, K Johansson; *Ladulaas Kliniska Studier,Skene:* K Berndtsson-Blom, R Andersson. K Davidsson, I Wiklund; *me3plus Clinical Trials, Göteborg:* C-P Anderberg, D Curiac, F Enqvist, D Norberg, E Dahl, H Åkesson; *Bollnäs hälsocentral, Bollnäs:* U Sundström, H Frostebro, M Broman; *Hässleholms Sjukhus, Hässleholm:* I Timberg, M Stjernberg, P Wikström; *Blekinge Sjukhuset, Karlshamn:* S Jensen, M Främby, A Glaas, C Olsson; *Oskarshamns Sjukhus, Oskarshamn:* U Mathiesen, K Fehling, U Robertsson; *Stockholm Heart Center, Stockholm:* A G Olsson, P Löf, S

Gunvarssdotter, L Bastani. **Merck clinical liaison** D Kush, J Anderson, J Fable. **Northwest Lipid Metabolism And Diabetes Research Laboratory, Seattle, USA** S Marcovina, H Gong.

Supplementary Table 1

Baseline characteristics of participants included in comparisons of **A:** summary PIL versus standard PIL; and **B:** modified PIL versus standard PIL.

A

Characteristic		Summary PIL<br>%	Standard PIL<br>%
Age (years)	<60	3%	3%
	≥60 <70	22%	23%
	≥70	74%	73%
Sex	Male	68%	68%
	Female	32%	32%
Prior disease	MI	42%	43%
	Stroke	24%	23%
	PAD	21%	21%
	Diabetes	26%	26%

MI: myocardial infarction; PAD: peripheral arterial disease

B

Characteristic		Modified PIL<br>%	Standard PIL<br>%
Age (years)	<60	6%	6%
	≥60 <70	23%	23%
	≥70	71%	71%
Sex	Male	68%	68%
	Female	32%	32%
Prior disease	MI	37%	36%
	Stroke	20%	20%
	PAD	23%	24%
	Diabetes	29%	28%

MI: myocardial infarction; PAD: peripheral arterial disease

Appendix 1: Original Participant Information Leaflet

See next page.

PARTICIPANT INFORMATION LEAFLET

HPS2-THRIVE

Treatment of HDL to Reduce the Incidence of Vascular Events

INVITATION TO JOIN A LARGE
MEDICAL RESEARCH PROJECT

A randomised study of ER niacin/laropiprant for the prevention of cardiovascular events in patients with vascular disease

You are being invited to take part in a research study. Please take time to read the following information carefully and discuss it with friends or relatives if you wish. You are entirely free to decide whether or not to take part in this trial. If you choose not to take part, the standard of care given by your own doctors will not be affected.

- Part 1 tells you the purpose of the study and what will happen to you if you wish to take part.
- Part 2 gives you more detailed information about the conduct of the study.

If there is anything that is not clear, or if you would like more information, please call Freefone (0800 585323) or speak to the local HPS2-THRIVE research nurse.

Please see reverse for a summary of the main information contained in this booklet.

Coordinated by:
Clinical Trial Service Unit, University of Oxford
Email: thrive@ctsu.ox.ac.uk
Website: www.ctsu.ox.ac.uk/hps2-thrive

Reference: HPS2-THRIVE Protocol V4.0_2008-04-18

Cholesterol, heart disease and strokes

People who have already had a circulatory problem such as a heart attack or stroke are at increased risk of developing further circulatory problems. One of the causes of this circulatory disease is having too much LDL (bad) cholesterol in the blood. We know that lowering this bad cholesterol in the blood with drugs such as statins reduces the risk of a heart attack or stroke. Everybody in this study will therefore be given tablets to lower their bad LDL cholesterol. However, despite effective LDL-lowering treatments, some people still suffer recurrent heart or circulatory problems. This study hopes to find a way of reducing these risks even further.

Does raising good cholesterol with niacin prevent heart attacks and strokes?

As well as LDL (bad) cholesterol there is also HDL (good) cholesterol in the blood. In general, people with higher levels of good HDL cholesterol have fewer heart attacks, strokes or circulatory problems than people with lower levels. Niacin is a medication that increases the amount of good cholesterol in the blood. However, although niacin has been in use for more than 50 years, it is still not clear whether it prevents heart attacks and strokes. Part of the difficulty has been that patients treated with niacin frequently develop flushing (reddening) of the skin, and sometimes other side-effects, making it difficult for people to take their tablets regularly. Extended release (ER) preparations of niacin reduce these side-effects but do not completely avoid them.

A new treatment, laropiprant (formerly MK-0524), has been developed which reduces the flushing caused by niacin. It works by blocking the dilatation of the blood vessels in the skin responsible for the flushing. Therefore laropiprant has been combined in a single tablet with extended release (ER) niacin to make it easier for people to take these treatments regularly. This combination is known as ER niacin/laropiprant (formerly MK-0524A).

What HPS2-THRIVE hopes to answer and how

The aim of HPS2-THRIVE (Treatment of HDL to Reduce the Incidence of Vascular Events) is to find out whether long-term treatment with ER niacin/laropiprant in people who have survived a heart attack, stroke or some other circulatory problem produces benefits by raising HDL (good) cholesterol. It is hoped that this will prevent heart attacks, strokes or the need for arterial bypass procedures (known as revascularisation) but this is as yet unknown.

In order to find out if ER niacin/laropiprant is beneficial, people taking part in the study will be put into 2 groups. This will be done randomly and one group will receive active ER niacin/laropiprant tablets and the other will receive a dummy version which looks and tastes identical. Neither you, nor your nurse or doctor will know whether you are taking the active or the dummy version. At the end of the study the outcome in the 2 groups will be compared. This type of research is called a randomised double-blind study.

Providing effective LDL-cholesterol lowering for all participants

In HPS2-THRIVE everyone will also be asked to take one tablet daily to lower their LDL cholesterol.

This will be either simvastatin alone or a single tablet containing the combination of simvastatin and a drug that also lowers cholesterol called ezetimibe. Which type you are provided with will depend on your previous statin treatment (if any), and your level of LDL (bad) cholesterol. If simvastatin 40 mg alone does not lower your LDL cholesterol enough, you will be given the combination tablet containing ezetimibe 10 mg plus simvastatin 40 mg (known as ezetimibe/simvastatin 10/40 mg).

All participants will therefore have effective LDL cholesterol-lowering treatment which aims, if possible, to get LDL cholesterol levels to below about 2.0 mmol/L (the currently recommended target). Both simvastatin and ezetimibe have previously been shown in large trials to be safe and effective treatments for lowering LDL cholesterol levels in people with circulatory problems.

Why have I been chosen?

HPS2-THRIVE will involve a total of 20,000 men and women. About 7,500 will be from the UK, plus a further 12,500 from Scandinavia and China. Like you, they are being invited to take part because they have already had some circulatory problem. This invitation has been sent either because you have participated successfully in previous trials (such as the Heart Protection Study) or, with the permission of your own doctor, because your medical records suggest you might be suitable for the study.

Do I have to take part?

If you are suitable, it is up to you whether you take part. If you do decide to take part in this study, you would, of course, be free to withdraw from the study treatment at any time without necessarily giving any reason (and without adversely affecting the medical care you can expect from your own doctors). In particular, at the end of the first few months, you will have the chance to withdraw if you have any second thoughts about being in the study or have any problems with study treatments.

What will happen to me if I take part

Everyone taking part will have agreed to do so voluntarily, knowing that it may involve them in taking study treatments for at least 4 years. If you agree to take part, the study nurse will need to see you in the clinic 3 or 4 times in the first 6 months and then 6-monthly. You will be asked some questions about your medical history, have a blood sample taken and measures of your height, weight and blood pressure. If you are eligible and wish to enter the study, you will be asked to sign a Consent Form and be given a copy to keep.

If you are not on a statin for your cholesterol you will be given study simvastatin 40 mg daily to take regularly. Alternatively, if you already take a cholesterol-lowering statin tablet, you will be asked to stop this and take study simvastatin 40 mg daily instead (with or without ezetimibe depending on the dose of statin you have been taking previously). If you are initially given simvastatin 40 mg alone and this is found not to lower your cholesterol enough, it will be changed to the combination of simvastatin and ezetimibe at the second visit.

For your early visits it will be helpful if you could fast. This means avoiding any food or drink (other than water) for at least 4 hours before the clinic. This helps with the reliability of the blood tests and these visits would be scheduled at a time of day to make it easy for you. At these visits the nurse will measure your cholesterol and give you these results.

Early in the study, you will be given a treatment-pack containing active ER niacin/laropiprant tablets. You will be asked to take one tablet a day for the first 4 weeks increasing to two tablets a day for the following four weeks. The study tablets are to be taken with food in the evening or at bedtime. After 8 weeks of taking laropiprant tablets plus the LDL-lowering tablet you would visit the clinic again. You would then decide if you are willing to continue taking study tablets for at least 4 years. The purpose of this part of the study is to make sure that the ER niacin/laropiprant agrees with you. If you do get side-effects at this stage, it may not be appropriate for you to continue in the study.

If you decide to continue, you will be given a further supply of study tablets and an appointment for 3 months time. Throughout the rest of the study, there will be three tablets to be taken each day, the LDL-lowering tablet (simvastatin or ezetimibe/simvastatin) and two tablets which contain either active ER niacin/laropiprant or a similar looking inactive dummy substance called a “placebo”. Whether or not a participant receives active or dummy tablets (placebo) will be determined randomly (like tossing a coin). Each participant will have a 50% chance of receiving active ER niacin/laropiprant combination tablets and a 50% chance of receiving placebo (“dummy”) tablets. The type of study treatment being taken will not generally be known by you or your doctor. This information will be known only by certain staff at the coordinating centre in Oxford, but it would be made available to your doctor if this were ever medically necessary. This design helps ensure that reliable information will be obtained about the effects of these potentially important treatments.

After the first 6 months you would need to attend for an appointment every 6 months. It will not be necessary to fast for these clinic visits. At every visit (each lasting 20-30 minutes), the study nurse will ask how your health has been since the last appointment, take a blood sample and provide you with more study treatment as required. In the unlikely event of a problem, we may need to ask you to return for an extra visit.

Expenses

You will be offered travel expenses for attending the study clinics.

What do I have to do?

If you agree to enter the study you will be asked to take study tablets daily for at least the next 4 years. The number of extra tablets will increase to 3 in the early stages (but you will no longer need to take your prescribed ‘statin’ if you were taking one). One tablet will be to control the LDL (bad) cholesterol and the other 2 will be to increase the HDL (good) cholesterol. Only half the participants will get active HDL raising tablets, but everyone will receive active tablets to control their LDL cholesterol.

For the study to get reliable results it is important that as many people as possible continue taking the study tablets during the whole study. If you think you may find that difficult it may be best if you do not join the study.

Similarly, if you think that you may have difficulty attending the study clinic appointments then it is probably best that you do not enter this trial. If you do decide to enter, your GP will be informed.

If you do join the study, we would like you to attend the specially set up study clinics 3-4 times in the first 6 months and then every 6 months. These visits will be extra to any visits to the doctor you may need. Occasionally people may be asked to attend for extra visits if you have a problem with the study tablets. If you are unable to attend on any occasion, or if you have any other queries or

questions about the study you can telephone (Freefone 0800 585323) and either rearrange your appointment or talk to one of the study doctors or nurses.

What is the drug that is being tested?

The drug being tested is a combination of niacin and laropiprant, the combination is called ER niacin/laropiprant. Laropiprant is a new drug which has been well tolerated in previous smaller clinical studies. Laropiprant is being used in this study to reduce any flushing that may occur with niacin. Prior to starting this study about 3000 people have received doses of either laropiprant or ER niacin/laropiprant. As it is a new combination drug, you will be asked to report any adverse effects you think may be due to the treatment.

What are the side-effects and risks of taking part?

Laropiprant is not known to have any particular side-effects, but it is possible there may be some, as yet unknown, side-effects.

Niacin is a drug that has been used to treat cholesterol for over 50 years. However, its use has been limited by skin flushing (defined as a feeling of warmth, redness, itching and/or tingling) as well as other side effects it can produce. Even though laropiprant should reduce any flushing due to the niacin, some people (20-30% in the first week) taking ER niacin/laropiprant may still experience flushing episodes. These episodes are more likely to occur within the first few days of starting treatment and then usually disappear by the end of the first week. They may then recur if you miss the tablets for a few days and then restart. Although occasionally unpleasant, these flushing episodes are not dangerous.

Some people may also experience other side-effects due to niacin, including: gastrointestinal symptoms (tummy upset, nausea or diarrhoea); headaches; skin rashes and rarely allergic reactions. Occasionally niacin can also cause liver problems (fewer than 1 in 100 patients) and this will be monitored by a regular blood test every time you come to the clinic. These liver problems usually resolve when treatment with niacin is stopped but rarely can make people unwell. Niacin can also increase blood sugar levels particularly in people with diabetes. The significance of such changes is unclear, but will be monitored during the study.

The simvastatin and ezetimibe are generally well tolerated although occasional side-effects have been reported. Very occasionally they can cause a muscle problem called ‘myopathy’ which causes muscle pain and/or weakness with abnormal blood tests, but this is rare (typically less than 1 in 10,000 affected per year among people of European origin but, information from the study so far shows rates of about 1 or 2 per 1000 per year in Chinese people). The risk may be increased slightly by taking additional niacin. **If, after joining the study, you were to develop some unexpected symptoms – in particular soreness or weakness of your muscles which is not the result of exercise or some other activity – you should to contact your study nurse, or one of the doctors at the coordinating centre (24 hour Freefone 0800 585323) in order to obtain advice**.

Effects of other treatments on taking part?

Certain medications when taken with simvastatin can increase the risk of the muscle side-effects. The most common of these are the antibiotics erythromycin and clarithromycin, but certain other drugs should also be avoided with study treatment including: fibrate cholesterol-lowering tablets,

verapamil, amiodarone, ketoconazole and itraconazole (your GP should be aware of this). People already taking these tablets will not be suitable to join the study. Also, if you have a history of cancer, other than skin cancer, in the last 5 years you would not be eligible for the study. People taking warfarin tablets are able to join the study but may need some extra anticoagulant (INR) checks when starting or stopping study treatments. Other tablets which people with a history of heart or circulatory problems commonly take are not known to interact with study treatments.

What are the possible benefits of taking part?

We hope that both the study treatments may help you by reducing the risk of a heart attack or stroke, however, this cannot be guaranteed. The information we get from this study may help us to treat future patients with heart disease better, and, if successful, may help to prevent many thousands of heart attacks, strokes and bypass procedures around the world.

What happens at the end of the study?

When the research study finishes we will inform you and your GP of the study results. Based on these, you will then be able to decide whether or not you should take ER niacin/laropiprant regularly. After the study finishes we will no longer continue to provide study medication for you. But, if the study results suggest you would benefit, your GP should be able to prescribe the treatments. We will also publish the study results in a professional medical journal as soon as possible after the study finishes. You would not be identified individually in any published report.

What if there is a problem?

Any complaint about the way you have been dealt with during the study or any possible harm you might suffer will be addressed. The detailed information on this is given in Part 2. The contact number for complaints is Freefone 0800 585323.

Will my taking part in this study be kept confidential?

Yes. All the information about your participation in this study will be kept confidential. The details are included in Part 2.

Contact Details

Any questions about the study should be directed to the coordinating centre in Oxford either by telephone
(24-hour Freefone service: 0800 585323)

or by post to:
HPS2-THRIVE, CTSU, Richard Doll Building, Old Road Campus, Roosevelt Drive, Oxford, OX3 7LF.

Alternatively you can e-mail us on thrive@ctsu.ox.ac.uk
This completes Part 1 of the Information Sheet.

If the information in Part 1 has interested you and you are considering participation, please continue to read the additional information in Part 2 before making any decision.

Part 2

What if new information becomes available?

Sometimes during the course of a research project relevant new information becomes available about the treatment that is being studied. If this happens we will tell you and your own doctor about it, and you can discuss whether you want to continue in the study. A study doctor is available through the 24-hour Freefone service (0800 585323) if either you or your GP need to discuss any new information.

What will happen if I don't want to carry on with the study?

You are free to withdraw from study treatment or from follow-up at any time. However, in order to obtain results that are as reliable as possible we would like to keep in contact with you at least by telephone to find out your progress. If you decide to stop the study treatment, we shall ask you to continue attending the study clinic if at all possible.

What if there is a problem?

If you have a concern about any aspect of this study, you should ask to speak with the researchers who will do their best to answer your questions **(24-hour Freefone service: 0800 585323)**. If you remain unhappy and wish to complain formally, you can do this through the NHS Complaints Procedure. Details can be obtained from the hospital.

In the unlikely event of you being harmed as a result of taking part in HPS2-THRIVE, insurance cover is provided by Merck & Co., Inc. who provide the study medication. Compensation for any injury caused by taking part in the study will be provided in accordance with the guidelines of the Association of the British Pharmaceutical Industry (ABPI). Compensation would be paid where the injury probably resulted from your taking the study drugs (or from any test or procedure) in line with the protocol. Any payment would be without legal commitment. We would not be bound by these guidelines where the injury resulted from a drug or procedure outside the trial protocol or if the protocol was not followed. In addition, you would retain the same rights of care as any other patient treated in the National Health Service.

Will my taking part in this study be kept confidential?

Information collected about you for the study will be entered directly onto a computer where it is stored securely, using encryption. This information will then be transferred to the central coordinating office at Oxford University where it will be stored long-term on computers protected by firewalls and in a secure building. In the central databases, personal information is stored separately from study information to which it is linked by a unique number. Access to study information is restricted to authorised study personnel on a need to know basis, and is controlled by usernames and passwords.

The coordinating centre would seek information from participants' own doctors and from NHS and other central registries about any serious illnesses (such as heart attacks, strokes, cancers etc) that occur (this requires patient identifiable information to be sent to these bodies). All information received would be used, in confidence, only for medical research purposes and for routine

regulatory and audit purposes. Nurse monitoring staff from the coordinating centre in Oxford may occasionally ask your permission to be present during your clinic visit to ensure procedures are being properly followed. Authorised people from regulatory agencies, the drug company and NHS bodies may look at the study information to ensure that the study is being carried out correctly but will be bound by rules of confidentiality.

Involvement of the General Practitioner/Family doctor (GP)

Your GP will be notified of your participation in the trial. Other medical practitioners not involved in the research who may be treating you should be made aware of your participation in this trial. You will be provided with a small card to carry giving details of the study. Your GP may be asked for additional medical details or given feedback on study findings.

What will happen to any samples I give?

Blood samples taken in the clinic will be used to check cholesterol levels, for safety checks and for central storage for trial related measures. A liver blood test will be done at every visit and if there are possible muscle problems a muscle blood test will also be done. Samples sent to the central laboratory are identified by a unique number linked in the computer to other study information.

You would also be asked if you are willing to allow us to store samples of your blood and urine for future analyses (including of your genes). This would be entirely optional and you would receive more information and a separate leaflet about this if you decide to take part in the trial.

Will any genetic tests be done?

This would be an entirely optional part of the trial and you would receive more details about this and asked to sign an additional consent form if you decide to take part.

What will happen to the results of the research study?

It is intended to publish the results of the research study in the appropriate scientific journal. No individual participant would be identified in any report or publication.

Who is organising and funding the research?

HPS2-THRIVE has been designed, and is coordinated, by Oxford University's Clinical Trial Service Unit. It involves the collaboration of many doctors and nurses around the country as well as in Scandinavia and China. The study design has been reviewed and agreed by independent Research Ethics Committees, which include people from outside the medical profession. An independent Data Monitoring Committee will review various outcomes among participants during the study, and will inform the organisers if any important new information has emerged that needs to be provided to participants and their doctors.

Packaged study treatment has been provided free by Merck & Co., Inc. who also provide a grant to Oxford University to run the study. The study is, however, conducted independently of the pharmaceutical company who have no say in its day-to-day running.

Who has reviewed the study?

This study was given a favourable ethical opinion for conduct in the NHS (or private sector) by the Thames Valley Multicentre Research Ethics Committee.

Please keep this information leaflet for your own records.

THANK YOU FOR TAKING THE TIME TO READ THIS SHEET

Summary of invitation to take part in HPS2-THRIVE

- Having circulatory problems increases the risk of subsequent heart attacks and strokes
- Statins and ezetimibe lower LDL (bad) cholesterol, and this benefits people who have survived a heart attack or stroke. Everyone in this study will be given LDL-lowering treatment
- Niacin raises HDL (good) cholesterol but side-effects include flushing of the skin
- ER niacin/laropiprant contains extended release niacin combined with laropiprant to reduce the flushing, and make the niacin easier to take.
- Half of those taking part in the study will be given active ER niacin/laropiprant and half will be given a dummy (placebo) version. Neither you nor your doctors will know which, and this will be decided randomly (like tossing a coin)
- The purpose of HPS2-THRIVE is to find out whether raising HDL cholesterol with ER niacin/laropiprant prevents heart attacks and strokes in people with circulatory problems who are already on treatment to lower LDL-cholesterol
- If ER niacin/laropiprant is shown to be safe and effective for people with circulatory disease, then its widespread use could lead to the prevention of many thousands of heart attacks and strokes and the saving of many lives
- With your help we can answer this question reliably with HPS2-THRIVE

If you have any questions about the study then please contact the coordinating centre on: Freefone 0800 585323

THANK YOU FOR YOUR HELP

Appendix 2: Invitation letter with one-page trial summary

See next page.

Attachment: T15-2A

Local Trust headed paper

T15-2A: Letter to Patient invitation to participate (patient identified from hospital records)

Department of General Medicine
Ambridge Hospital
Ambridge Hospitals NHS Trust

CTSU coordinating centre
Freefone: 0800 585323
Email: thrive.ctsu.ox.ac.uk

[Date]

[Patient address]

Dear [title] [Patient Name]

HPS2-THRIVE: Treatment of HDL to Reduce the Incidence of Vascular Events

I am writing to invite you to consider taking part in a clinical trial. HPS2-THRIVE is a study of the prevention of heart attacks and strokes in people with circulatory problems. The purpose of this study is to assess whether raising HDL cholesterol (the good sort) with niacin is useful for preventing heart attacks and strokes in people with circulatory disease. Niacin, which increases HDL cholesterol, has been available for many years, but its use has been limited by side-effects particularly flushing of the skin. It has now been combined with a new drug (laropiprant) which reduces this flushing making it easier to tolerate. It is known that people with naturally higher levels of good HDL cholesterol have fewer heart attacks and strokes. It is hoped that raising HDL cholesterol with these tablets will be beneficial, but this is not yet known.

This invitation has been sent to you - with the knowledge of your own doctor – since your medical records suggest you have had circulatory problems, and therefore might be suitable for this study. Please read the enclosed information. If you think you would be suitable, it is then up to you whether or not you would like to take part.

The HPS2-THRIVE clinic is held in the Long Building (block 26), Ambridge Royal Infirmary. **A provisional appointment with the local study nurse has been made for you on:**

Friday, 20 November 2006 at 2.00 p.m.

Please let us know as soon as possible whether or not you would like to attend this appointment. You can do this by telephoning free of charge on 0800 585323 (Monday to Friday, 9 a.m. to 5 p.m.) or by returning the enclosed form in the reply-paid envelope. If the appointment is not convenient then you can change it when you call us. Please arrive 15 minutes prior to your appointment time as there will be additional reading material available at the clinic.

PTO

We hope you will be happy to participate in HPS2-THRIVE. It would help us if you could bring a list of your prescribed medicines to the clinic and, if possible, avoid eating or drinking anything except water/black tea or coffee (without sugar) for at least 4 hours before you appointment. If you have any questions regarding the study you may telephone the study co-ordinators (Dr Jane Armitage or Dr Martin Landray) on Freefone 0800 585323. If you wish to bring someone with you such as your spouse or a friend, they would be most welcome.

Yours sincerely,

Dr Jack Black
Cardiology Department, Ambridge Royal Infirmary

Enc: Reply Form
Freepost envelope
Information sheet

Summary of HPS2-THRIVE

- Having circulatory problems increases the risk of subsequent heart attacks and strokes.
- Statins and ezetimibe lower LDL (bad) cholesterol, and this benefits people who have survived a heart attack or stroke. Everyone in this study will be given LDL-lowering treatment.
- It is known that people with naturally higher levels of good HDL cholesterol have fewer heart attacks and strokes. Niacin raises HDL (good) cholesterol but side-effects include flushing of the skin.
- ER niacin/laropiprant contains extended release niacin combined with laropiprant to reduce the flushing, and make the niacin easier to take.
- Half of those taking part in the study will be given active ER niacin/laropiprant and half will be given a dummy (placebo) version. Neither you nor your doctors will know which, and this will be decided randomly (like tossing a coin).
- Participation in the study will involve taking study treatment for 4 to 5 years. In the first 6 months this will involve 3 or 4 trips to clinic to see the study nurse and after this, appointments will be every 6 months.
- The first few months of the study will be a 'run-in' period in which you will be given active study treatment. During this time, you can see how you get on with the study treatment and consider whether you wish to commit to it for the next 4 to 5 years.
- Each visit will involve a blood test, answering some questions about your health and being given some tablets. Once fully established on the trial you would be asked to take 3 tablets daily: one to lower the bad LDL cholesterol and two trial tablets which may increase the good cholesterol or be dummy tablets.
- The purpose of HPS2-THRIVE is to find out whether raising HDL cholesterol with ER niacin/laropiprant prevents heart attacks and strokes in people with circulatory problems who are already on treatment to lower LDL-cholesterol.
- If ER niacin/laropiprant is shown to be safe and effective for people with circulatory disease, then its widespread use could lead to the prevention of many thousands of heart attacks and strokes and the saving of many lives.
- With your help we can answer this question reliably with HPS2-THRIVE.

If you have any questions about the study then please contact the coordinating centre on:
Freefone 0800 585323

Thank you for your help

Appendix 3: Modified Participant Information Leaflet

See next page.

THRIVE

WORKING TO REDUCE HEART ATTACKS AND STROKES

….an invitation to join a health research study.

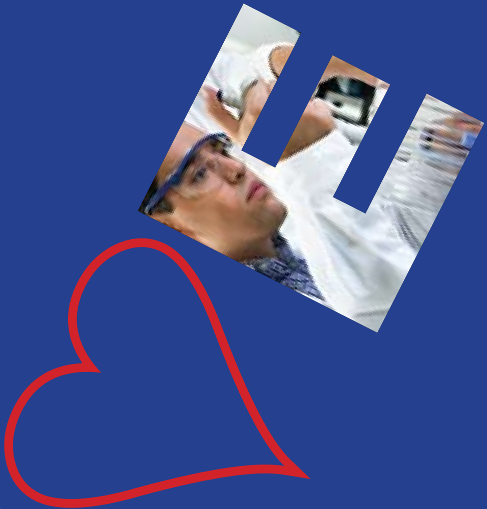

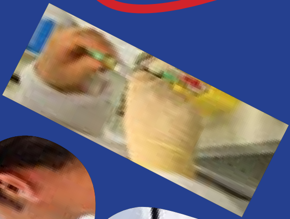

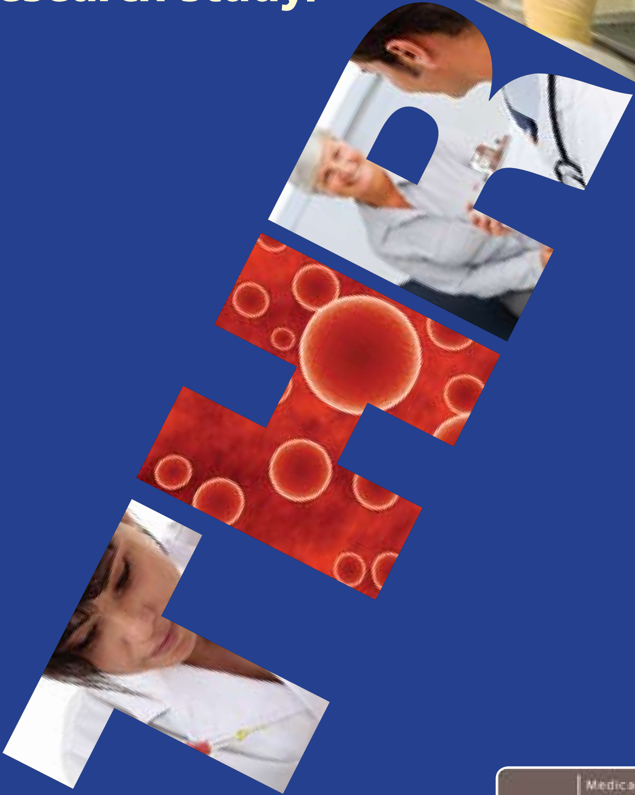

MRC Medical Research Council

HEART UK

OXFORD

Questions about the study should be directed to the coordinating centre in Oxford

By phone:
24-hour Freefone service:
0800 585323

By post:
HPS2-THRIVE, CTSU,
Richard Doll Building,
Old Road Campus, Roosevelt Drive,
Oxford, OX3 7LF

By email:
thrive@ctsu.ox.ac.uk

Or visit our website:
www.thrivestudy.org

Did you know there is such a thing as **good** cholesterol (and we don’t just mean low levels of bad cholesterol)? And the more you have, the better it is for you?

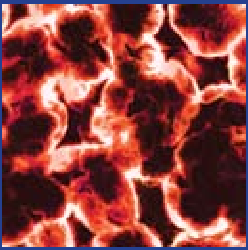

Cholesterol, heart attacks and strokes

If you have had a heart attack or stroke, you will know about cholesterol and the need to keep its level low. It can be a struggle, but a good diet, plenty of exercise and statins can help.

But did you know that there is a good type of cholesterol? And the more good cholesterol you have, the better – because it may help to prevent further heart attacks and strokes?

THRIVE: what is the purpose?

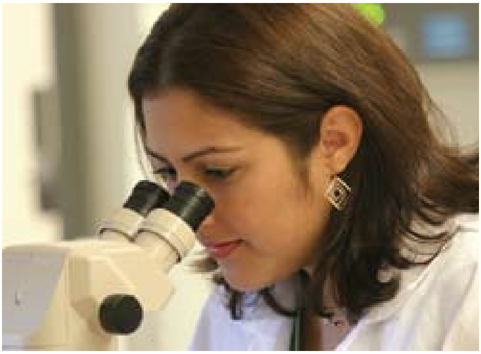

THRIVE is a study to see if boosting levels of good cholesterol can save more lives. THRIVE is doing this by testing a treatment called niacin, which raises levels of good cholesterol in your blood. Everyone taking part in THRIVE will also receive tablets, called statins, to lower their bad cholesterol. If you are taking statins already you can still join this study.

Why me?

You are being invited to take part in this study with the knowledge of your hospital consultant. Your hospital records suggest you may be suitable. We have also written to your GP to tell them that some of their patients are being asked to take part. If you agree to participate your GP will be informed.

You can take part whether or not you are already taking a statin to reduce your bad cholesterol. This is because this study is not looking at statins. Statins have been tried and tested successfully in similar studies over many years – to provide the evidence that they are safe and help protect people from heart attacks and strokes.

Who is running the study?

THRIVE is being led by medical scientists at Oxford University who carried out the important Heart Protection Study (HPS). This study showed conclusively how effective statins were at saving lives. That is why THRIVE is sometimes known as HPS2-THRIVE.

Do I need to take part?

You do not have to take part in this study. It is entirely your decision. But if you did help, many millions of people from around the world might benefit from this research in years to come.

Travel expenses

A contribution towards the cost of travelling to your THRIVE appointment can be provided. Make sure you ask at the clinic.

What happens to me if I take part?

Getting started

Your continued good health is the prime concern of the trained medical staff involved with the THRIVE study. They will monitor your health at regular intervals throughout the study. Your GP will be informed if you decide to join the study.

At your first visit to the THRIVE clinic a trained researcher (usually a nurse) will ask you about your medical history. The researcher will take your blood pressure and a sample of blood. They will explain the study to you. If you are interested you will be asked to sign a form agreeing to take part. Over the course of the next few weeks you will have the chance to try out the tablets. You will also be measured and weighed. You will then be asked to make a commitment to the study long-term. You can withdraw from the study at any time. If you do stop taking the tablets after committing to the study long-term, we would still like to keep in touch with you, if possible. Ideally this means still coming to the study clinic, if this is not possible then by phone.

Further information about the study is available from the THRIVE clinic nurses.

For the first few visits it is helpful if you avoid any food and drink – other than water and any usual tablets – for at least 4 hours before your appointment. If you have diabetes and would like advice about fasting please call Freefone 0800 585323.

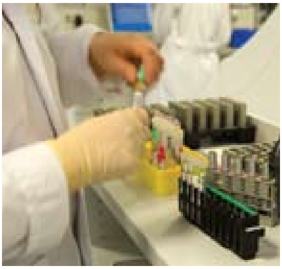

Increasing good cholesterol

The tablets being tested contain niacin and a compound called laropiprant. It is known that niacin increases levels of good cholesterol in the blood. Doctors hope that this will reduce the number of heart attacks and strokes, but they do not know this for certain. The laropiprant will reduce the side effects that people taking niacin have had before. People have experienced flushing (reddening) of the skin, which laropiprant is designed to prevent.

'It is known that niacin can increase levels of good cholesterol'.

In the first few weeks you will be asked to take one of these tablets each day. After 4 weeks, this will increase to two tablets a day.

Lowering bad cholesterol

Everyone taking part in THRIVE will also be given a statin to reduce their bad cholesterol. You will be asked to take a statin called simvastatin. This may mean that you have to change from your current treatment. However, all statins work in very much the same way, and this should not cause you any problems.

The amount of statin-based treatment you receive on the THRIVE trial will be at least as strong as your current treatment.

Usually, this is just 40mg of simvastatin daily. In the first few months, trained specialist THRIVE researchers will monitor your levels of bad cholesterol to ensure that the statin treatment works satisfactorily. After that your doctor can keep an eye on your levels if they wish to.

This treatment aims to get your cholesterol to the current recommended target (below about 4.0 mmol/l).

You may be given a statin booster to help get your bad cholesterol down further, if necessary. This is a tablet combining simvastatin 40mg with ezetimibe 10mg. It is known as ezetimibe/simvastatin 10/40mg.

Simvastatin is provided free to you by the study team. Even if you decide eventually not to carry on with the study tablets, you can still keep taking these statins.

THRIVE researchers ask all participants to use the same statin so that the 'background' treatment to reduce bad cholesterol in all participants is the same. This helps the research scientists to know that any results they get from the trial to increase good cholesterol are 'real'.

Simvastatin and ezetimibe have been shown in large trials to be safe. They are effective treatments for lowering levels of bad cholesterol.

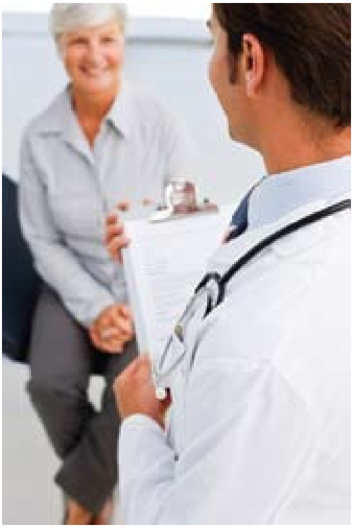

'People already taking statins can take part in this study'.

After two months

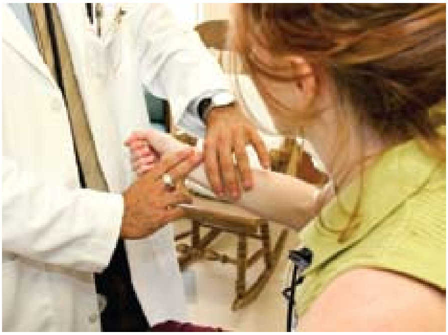

During the early part of the study you are provided with niacin, the treatment under examination, to check if it suits you. You also receive simvastatin, to reduce your bad cholesterol. The tablets are taken with food in the evening, or at bedtime.

You will be monitored so that the THRIVE nurses can be sure the niacin agrees with you, and that your bad cholesterol is under control.

The detail

After 8 weeks of trying the study tablets you will then be asked if you would like to continue with the study. If you agree, you will be asked to commit to the study for the next four years.

Everyone on the trial will continue with their statin treatment.

However, only half of the participants will receive the tablet containing niacin. The other half will be given a dummy drug, called a placebo. Scientists do not know which treatment is best, but this study should give the answer.

Whether or not you receive niacin is done randomly. This means that you have as much chance of receiving it, as you do in not receiving it (like the toss of a coin). You will not know which treatment you receive, nor will your GP or the THRIVE staff. However, this information would be made available to your doctor if this was medically necessary.

After 6 months

After the first 6 months, you will attend a 20-30 minute appointment every six months. A sample of blood will be taken and you will be provided with more treatment for the

Study Timetable

Duration: About 8 - 16 weeks, depending on the individual

First Appointment
When you provisionally agree to take part

↓

Duration: up to 8 weeks
During the early months, study nurses will check that your statin treatment is adequate and you can try out the niacin tablets.

Participants already on statins used in the trial may be able to miss out this stage.

Duration: 4 weeks:
Statin + one niacin tablet

↓

Duration: 4 weeks:
Statin + two niacin tablets

Study Timetable

Duration: About 4 years

One visit to the THRIVE clinic after 3 months and then an appointment every 6 months

Confirm long-term commitment to the study

Participants are selected randomly (by chance) to receive one of these treatments.
Doctors do not know which one is best.

Half of all participants receive a statin and niacin.

Half of all participants receive a statin and a dummy tablet called a placebo.

Study finishes
Results are published

next six months. You will not need to fast beforehand. With regular check ups from the THRIVE specialist nursing team, you can be assured of the best possible follow up care and attention.

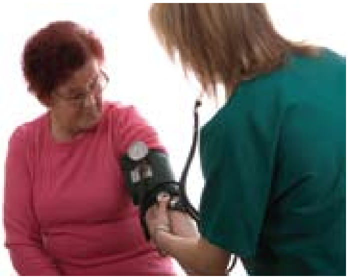

If you do choose to withdraw, it would be helpful if you would allow the study scientists to stay in touch with you, to see how you get on. If any problems emerge for you while you are on the study, your GP will be informed.

At each visit the THRIVE nurse will take a blood sample. This will be tested to check the liver. If you have muscle problems, a muscle blood test will be done. Sometimes the blood samples will be sent to the laboratory in Oxford for extra tests.

'It is important people stay in the study for as long as possible'.

What will I have to do?

For THRIVE to produce the best results, it is important that people stay in the study for as long as possible. We will ask you to commit to the study for at least four years. You will need to attend the THRIVE clinic several times in the first few months and then once every six months.

You will be asked to take a statin called simvastatin and either a treatment to raise your good cholesterol, or a dummy treatment called a placebo. Scientists do not know which treatment is best.

You will also be asked to allow your blood and urine to be stored for future analysis.

You can withdraw from the study at any time.

What is the treatment being tested?

A treatment to raise good cholesterol with a combination of niacin and laropiprant is being tested. Before this study about 3,000 people received this treatment. About 25,000 people in the UK, Scandinavia and China will take part in THRIVE. Already over 20,000 people have joined the study.

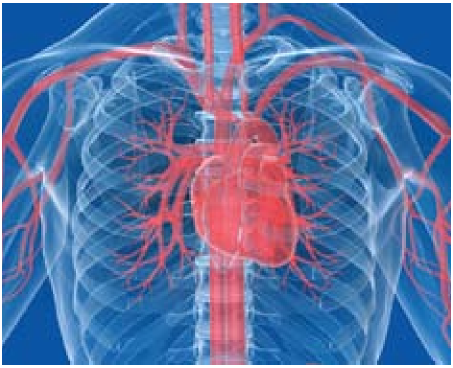

What are the benefits of taking part in this study?

You may be helping yourself, but you will most certainly be helping doctors and scientists improve treatment for people who have had heart attacks or strokes, or who may be at risk of having one. We hope that being on the study may help you by reducing the risk of having a heart attack or stroke. If successful, results from this study will help to prevent many thousands of heart attacks and strokes and bypass procedures around the world.

Are there any risks?

Most treatments have side effects which some people may experience and others do not. If you do experience any side effects while on the THRIVE study they will be noted, so that scientists can learn from you. You can withdraw from the study if you wish.

◆ **Laropiprant** is not known to have any side-effects. It may be that some emerge in this study, but doctors think this is unlikely. If common side-effects did emerge, you would be told about them;

◆ **Niacin** use has been limited because it can cause a feeling of warmth, redness and itching of the skin (flushing) in some people. Laropiprant ought to reduce this side-effect.

However, some people may still experience flushing in the first few weeks of taking the treatment. Though unpleasant, flushing episodes are not dangerous. Some people experience tummy upset, nausea, diarrhoea and rashes and rarely allergic reactions. Blood tests are used to monitor for a rare liver problem (that usually resolves when niacin is stopped). Niacin can also increase blood sugar levels, particularly in people with diabetes. However, researchers running THRIVE hope that people with diabetes will take part. Steps can be taken to control their blood sugar levels. The study may help to explain why niacin raises blood sugar in this way.

◆ **Statins** are generally very well tolerated. A rare side-effect is muscle pain and weakness.

There is nothing to suggest that stopping the tablets will cause you harm. If you do experience side-effects, you may choose, or be advised by your doctor, to stop the tablets provided by the study but you can usually continue to take your statins.

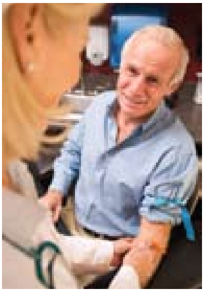

If you do experience unexpected symptoms after joining the study you can contact your THRIVE nurse, or a study doctor on Freefone 0800 585323.

Will my taking part be kept confidential?

Yes, absolutely. Information about you is entered on to a computer and stored securely. Oxford University is a world-leader in developing systems to ensure that information is stored safely for studies such as THRIVE.

The coordinating centre will ask for information from your doctors and from NHS and other central registries about any serious illnesses that you have. All information received is used in confidence only for medical research purposes and for routine regulatory and audit purposes.

Blood samples are sent to a laboratory at Oxford University for analysis. They are identified by a unique number linked in

the computer to other study information. In the laboratory they are not linked to your name.

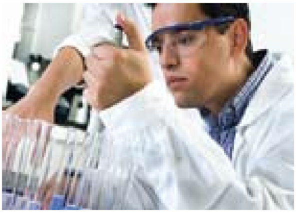

The information used for scientific analysis will not include any details that identify you.

What will happen at the end of the study?

The results will be published widely in health or scientific journals and be discussed at major conferences. Others will learn from the results which we hope will show that more lives can be saved by increasing good cholesterol. No individual participant will be identified in any report or publication. We will endeavour to inform participants and their GPs of the results, and any ensuing publicity.

'Results from the study will be published and help doctors improve the treatment of heart attacks and strokes'.

How is this study organised?

Scientists and doctors consider the questions being asked by THRIVE to be important, because they could improve treatment for people who have had, or may be at high risk of, a heart attack or stroke. THRIVE is built on the success of the Heart Protection Study which showed how useful statins are in saving lives.

Scientists at Oxford University, who are carrying out the project, have had to get permission from ethics committees to do so. These committees check whether the health question being asked is important enough to

warrant a study, and that the study is being carried out in an independent, honest and professional manner.

An independent committee also watches over the study and keeps an eye on results. This committee could halt the study early if important new evidence emerged that had an impact on the need for the study to continue.

THRIVE has been designed and is coordinated by Oxford University's Clinical Trial Service Unit (CTSU), one of the world's leading centres for this type of work. The study involves many heart doctors and nurses around the country and in Scandinavia and China.

THRIVE is lead by Professor Jane Armitage at CTSU, a qualified and practising doctor at an Oxford hospital. She has played an important role in previous studies that have shown the health benefits of statins.

Independent studies such as THRIVE are costly to run. Treatment for the study is provided free by Merck & Co., Inc. which also contributes to the cost of running the study, by a grant to Oxford University. Merck also provided some of the funding for the Heart Protection Study that provided clear evidence of the benefit of statin treatment to reduce heart attacks and strokes.

However, THRIVE is conducted independently of Merck, which has no say in the running of the project.

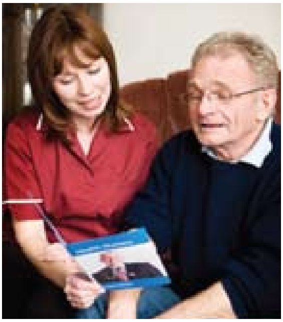

'THRIVE is testing niacin, not statins, to see if we can improve health further'.

What if there is a problem?

You retain all the usual rights of an NHS patient.

In the unlikely event of your being harmed by taking part insurance cover is provided by Merck & Co., Inc. Any compensation would be paid in accordance with the guidelines of the Association of British Pharmaceutical Industry (ABPI). Any payment would be without legal commitment.

If you have a concern about any aspect of the study you can speak with the researchers. They can be contacted on a 24-hour Freefone number: 0800 585323.

Thank you

Thank you for your interest in this study. More information is available from the THRIVE clinic nurses, by calling the telephone number above, or from the THRIVE web site: **www.thrivestudy.org**

If you would like to take part, travel expenses to and from the THRIVE clinic can be claimed. Our aim is to make your participation an interesting and worthwhile experience while helping us and others to improve the treatment of people who have had, or who may have, a heart attack or stroke.
